# Treating endothelial dysfunction with vitamin D in chronic kidney disease: a meta-analysis

**DOI:** 10.1186/s12882-018-1042-y

**Published:** 2018-09-25

**Authors:** Kristina Lundwall, Stefan H. Jacobson, Gun Jörneskog, Jonas Spaak

**Affiliations:** 1 Department of Clinical Sciences, Danderyd University Hospital, Karolinska Institutet, Stockholm, Sweden; 2 Department of Cardiology, Danderyd University Hospital, 182 88 Stockholm, Sweden

**Keywords:** Endothelial function, Flow mediated vasodilation, Cholecalciferol, Paricalcitol, Renal failure

## Abstract

**Background:**

Vitamin D deficiency is common in patients with chronic kidney disease (CKD), and is associated with endothelial dysfunction and cardiovascular disease. We performed a meta-analysis to assess the effect of vitamin D treatment on flow mediated vasodilation (FMD) in CKD patients.

**Methods:**

PubMed/Medline, Web of Science, Embase and Cochrane trials and reviews were searched systematically for randomized controlled trials (RCT:s) using any vitamin D compound, at any stage of CKD, with FMD as outcome. Fixed and random effects models were performed using the standardized mean difference effect size post treatment for each trial. Heterogeneity was assessed by I^2^ statistics.

**Results:**

4 trials were included, comprising 305 patients. One used both 1 and 2 μg for two intervention groups and was therefore split in two during the analysis. Patients in the included trials had a mean age of 44–65 years and were all in CKD 3 to 4. One study used cholecalciferol, the others all used paricalcitol as treatment. Study duration was 12–16 weeks. Intervention with vitamin D was associated with ameliorated FMD (STANDmean ES 0.78, 95% CI 0.55–1.01) in a fixed model. Heterogeneity was substantial (I^2^ = 84%). Secondary analysis with random model analysis also showed significant results.

**Conclusions:**

Short term intervention with vitamin D is associated with improvements in endothelial function, as measured by FMD. This indicates positive effects of vitamin D on vascular disease in CKD. Limitations of this meta-analysis are the small number of studies performed, and the short duration of intervention.

**Electronic supplementary material:**

The online version of this article (10.1186/s12882-018-1042-y) contains supplementary material, which is available to authorized users.

## Background

Chronic kidney disease (CKD) is a worldwide health issue, affecting 10–15% of the population with high costs both for patients and society [1, 2]. The main reasons for death is not, however, end stage renal disease, but more often cardiovascular events [2, 3]. The acknowledgement of kidney dysfunction as a strong risk factor for cardiovascular (CV) disease is highlighted in the European Society of Cardiology (ESC) [4] and the Kidney Disease Improving Global Outcome (KDIGO) guidelines [5]. Even so, treatment options to affect outcome are few, and evidence of these on cardiovascular hard end points are sparse.

Most CKD patients suffer a pronounced vascular disease, with endothelial dysfunction from early stages [6], and later on a marked vascular stiffening and arterial calcification [1, 3]. The reasons are multifactorial though with emphasis on chronic inflammation and disturbances in the hormonal mineral bone disorder (MBD) axis, with vitamin D deficiency, secondary hyperparathyreoidism (hPTH), high phosphate, and FGF-23 levels and downregulation of Klotho [1–3] [7].

Vitamin D has been shown to have anti-inflammatory and anti-oxidative properties ([8]). Vitamin D also down-regulates the expression of renin and has therefore gained interest as a possible treatment option in CKD [9, 10]. Meta-analyses from the last decade show that vitamin D affects residual albuminuria/proteinuria, on top of RAAS blockade [11–14] probably due to anti-inflammatory effects, such as downregulation of the TGF-beta pathway [13], downregulation of renin expressing genes [9, 10], and based on synergy with the AT1-receptor [15]. Glucose metabolism is another interesting area, where one meta-analysis shows positive effect on glucose control by treatment [16].

There has been some concern that active vitamin D compounds might cause a deterioration of renal function. Zhang et al. [17] performed a meta-analysis that showed higher creatinine levels with treatment, but no effect on eGFR when measured without creatinine, interpreted as no real effect on eGFR but probably higher creatinine due to an altered creatinine metabolism with active compounds [12]. These results are in line with other meta-analyses investigating the same area [12, 14].

Meta-analyses on cardiovascular risk and mortality show effect of treatment with vitamin D in CKD patients in observational studies [18, 19]. One meta-analysis [20] investigated the effect on cardiovascular endpoints in controlled trials, but could not show any benefit of treatment. These results may be questioned however, since none of the included studies had these endpoints as à priori primary or secondary endpoints, and the study durations varied from 3 weeks to 2 years.

There is a lack of interventional studies of vitamin D in CKD with sufficient power to answer questions on hard endpoints. In the absence of hard endpoints, surrogate markers of cardiovascular risk have been used in interventional trials, such as flow mediated vasodilation (FMD) since endothelial dysfunction precedes manifest vascular disease [21, 22]. In vitro data support the notion of a direct effect of vitamin D on endothelial function, with decreased oxidative stress and augmented levels of eNOS [23–25]. This makes endothelial function measured by FMD an interesting topic for a meta-analysis in the small, short duration studies performed in the area.

## Methods

### Study inclusion criteria.

This meta-analysis was made in accordance with the PRISMA guidelines and checklist. We included randomized controlled trials using a placebo or no treatment group as control. The population was restricted to CKD patients, in any stage of the disease, with or without diabetes mellitus. Intervention was considered treatment or supplementation with any vitamin D compound. Outcome was limited to FMD. Exclusion criteria were combined vitamin D and calcium treatment, or comparison to other vitamin D compounds (active versus precursor) or to calcimimetics, without a non-treatment control group.

### Data sources and searches

Together with two librarians specialized in data base searches, we performed a systematic search of available literature. To avoid too many negative results the search was set between year 2000 and 2018-03-22, since the cardiovascular protective effects of vitamin D were not investigated before that time. PubMed/Medline, Embase and Web of Science (WoS) as well as Cochrane reviews and Cochrane trials were searched in a systematic way. We used the MeSH-term for vitamin D as well as kidney disease and then all terms listed beneath, words from entry terms, and words found in relevant abstracts. Words used were checked against abstracts to make sure they were relevant. Search results were restricted to controlled trials and to the English language. Conference abstracts were included in the search. A full report of the search strategy, including information on software and special features, is available in the supplemental material.

### Data extraction and methodological study quality

Data was extracted by two blinded investigators, in accordance with a standardized extraction form, including terms as study length, number of participants, vitamin D compound and dosage, age, CKD stage, and other treatments (Table 1). There were too few studies to account statistically for interrater reliability, but the extraction forms were well matched and differences resolved by discussion between the authors. Methodological study quality was assessed by the Jadad Score [26], which give 1–5 points for blinding, randomization and the account of all screened and included patients. There were no remaining questions after data extraction. Therefore no further contact with authors was needed.

**Table 1:** Characteristics of included studies

Author	Zoccali (− 14)	Lundwall 2 μg (− 15)	Lundwall 1 μg (− 15)	Theti (− 15)	Kumar (− 17)
Country	Italy	Sweden	Sweden	USA	India
Duration	12 w	12 w	12 w	12 w	16 w
Sample size (nr)	89, analysis on 88	24, ITT	24, ITT	60, 55 completed, analysis on 46	120, analysis on 117
CKD stage	3–4	3–4	3–4	3–4	3–4
Treatment	paricalcitol	paricalcitol	paricalcitol	paricalcitol	Cholecalciferol
Dose	2 μg daily	2 μg daily	1 μg daily	1 μg daily	300.000 IU at baseline and after 8 week
Baseline 25 (OH) D(nmol/l)	35.5	65.1	66.7	1.25-OHD: 34.5 (pg/ml)	33.2
Age (mean)	62.5	65.0	68.6	62.5 (median)	44.2
ACEi/ARB (%)	N/A	80.6	80.6	69.1	67.5
Jadad score	4	4	4	3	5
Underlying condition/DM	N/A	Non-diabetic patients	Non-diabetic patients	Diabetic nephropathy	Non-diabetic patients
Outcome	FMD	FMD,PWV,echo,iontophoresis,microcirculation	FMD,PWV,echo,iontophoresis,microcirculation	FMD	FMD,PWV

*ITT* intention to treat, *ACEi* angiotensin converting enzyme inhibitor, *ARB* angiotensin receptor blocker, *FMD* flow mediated vasodilation, *PWV* pulse wave velocity, *Echo* echocardiography

### Data synthesis and analysis

We used standardized mean difference effect size (STANDmean ES) [27, 28] between treated patients and placebo/no treatment patients post treatment to assess the effect of vitamin D on FMD. We used the weighted standard deviation (SD) [28], when SD was presented in the article. In one case SD had to be estimated from range as (max-min)/4. In one article neither the exact value post treatment nor SD measures were presented in the article or the supplemental material and we then used the t-value, estimated from the *p*-value post treatment as recommended [28, 29]. The effect size for each study was calculated according to Hedges g [27, 28]. Standard error (SE) and 95% confidence interval (CI) were computed for all studies. A positive effect size indicated a result in favour of the treatment group.

Overall STANDmean ES for all included studies was assessed with a fixed effects model. Studies were weighted with the inverse variance weights technique giving more weight to studies with larger populations to allow higher impact to studies that yield a more precise estimate [27]. SE and 95% CI were calculated for the overall ES.

We also performed a random effects model, according to the DerSimonian and Laird estimate [27].

We computed correlations of effect sizes and study populations and funnel plots to investigate publication bias [27, 28].

To assess heterogeneity we calculated I^2^ statistics where < 50% was considered as minimal heterogeneity, 50–75% as moderate and > 75% as substantial heterogeneity [30, 31].

## Results

### Study selection

**Fig. 1:**
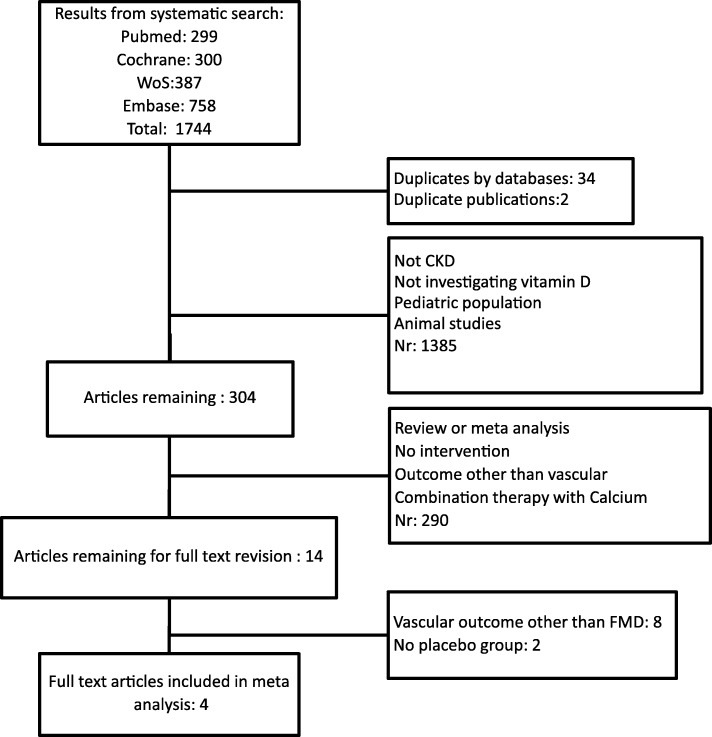
Flow diagram of the selection process

The screening and selection of articles was performed by two blinded investigators (author 1 and 4), and disagreements were resolved by discussion with the other authors. There were no remaining disagreements after the selection process. A total of 1744 articles were found searching the databases. After a first screening of title and abstract 304 articles remained. Of these, 14 were selected for full review. Four studies met the full inclusion criteria (Fig. 1). After discussion with all authors, one study was divided into two treatment groups, with 1 and 2 μg of paricalcitol, using the same placebo group as control. The 1 and 2 μg groups were regarded separately, thus resulting in 5 studies used in the meta-analysis. We did not find any conference abstract without a published article that was of relevance for our research question.

### Study characteristics

Study characteristics are presented in Table 1. Study size varied from 24 to 120 participants, and study duration from 12 to 16 weeks. Four studies examined the effects of treatment with paricalcitol in doses of 1 or 2 μg, and one study used cholecalciferol administered in two oral doses of 300,000 IU at baseline and after 8 weeks. Mean age was 59.9 years (mean) ranging from mean/median values of 44–65 years. All patients were in CKD stage 3–4.

### Quality assessment and risk of bias

The Cochrane Handbook 5–1 and the Jadad score were used to assess quality and risk of bias. Sequence generation, allocation sequence concealment, and risk of incomplete outcome data were assessed by the Jadad score. The 5 included studies had Jadad scores of 3–5, indicating median to high quality and an overall low risk of biased data. Selective outcome reporting was assessed during the screening and selection process, since we only used one outcome in the final analysis. None of the found studies reported FMD in the methodological section, but not in the results section. This together with our specified outcome indicate a low risk of selective outcome reporting.

Publication bias was assessed by rank correlation and by visual inspection of a funnel plot, and these results did not indicate publication bias. We also searched ClinicalTrials.gov without finding any unpublished material of interest for our inclusion criteria.

### FMD outcome

**Fig. 2:**
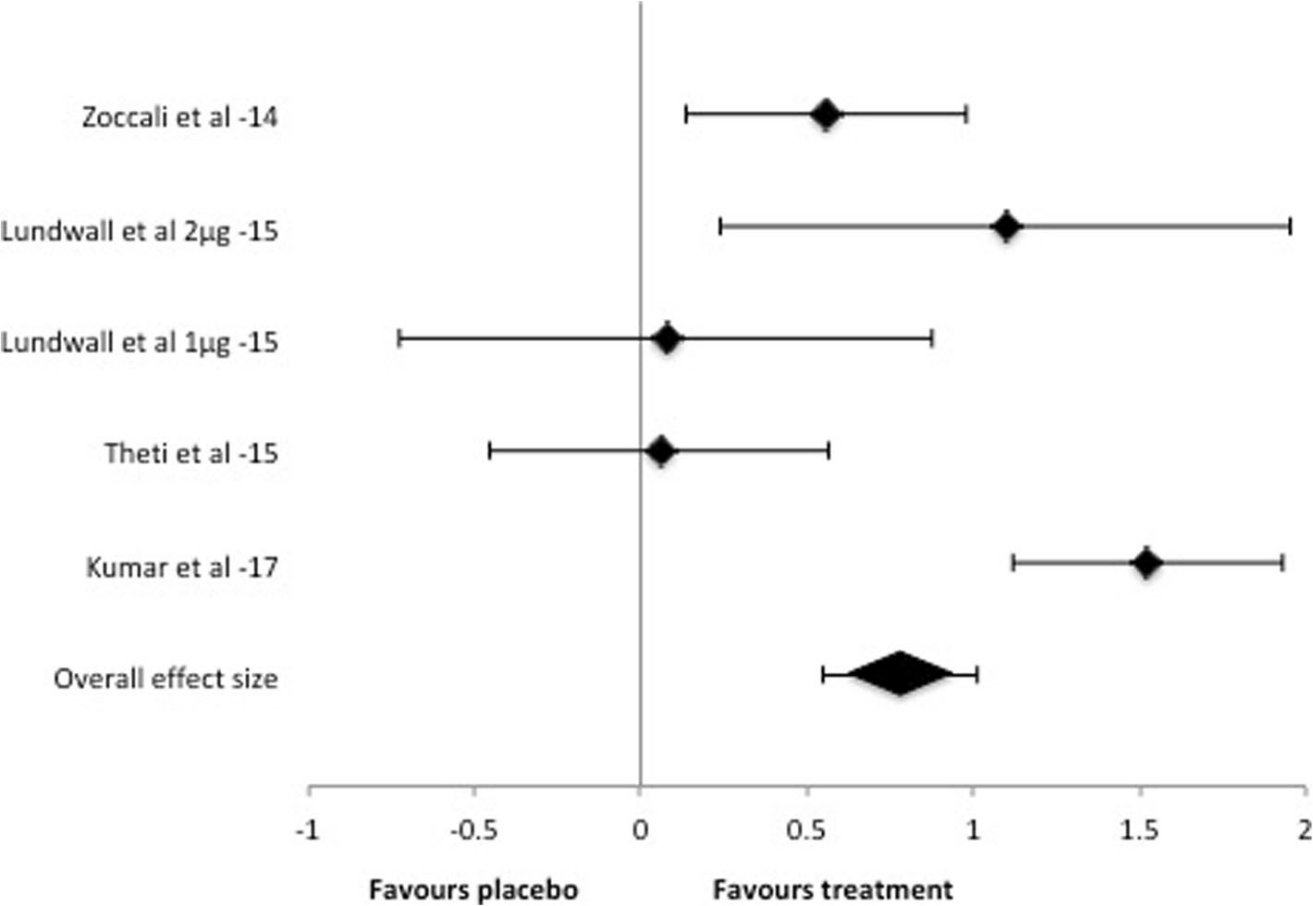
Forest plot of standardized effect size post intervention. A positive value indicates ameliorated FMD response in treated patients

Five studies with a total number of 305 patients were evaluated. There was no difference between the intervention group and the placebo group in measures of FMD at baseline in any study. Fixed effects model analysis of these studies indicated an overall effect of vitamin D treatment on FMD measures (STANDmean ES 0.78, 95% CI 0.55–1.01) (Fig. 2). Random effects model also showed a positive effect of treatment (STANDmean ES 0.67 95% CI 0.06–1.29). The heterogeneity across the included studies according to I^2^ statistics was substantial for the fixed model (I^2^ = 84%), but minimal for the random model (I^2^ = 0%). The number of studies was too few to perform a meta-regression to investigate the heterogeneity in the fixed model.

## Discussion

This meta-analysis of existing publications on intervention with vitamin D on measures of FMD shows significant effect on endothelial function, an important factor in vascular disease. Two studies using FMD as outcome were not included due to the lack of a control group. Chitalia et al. [23] showed positive effects using Cholecalciferol 300,000 IU, given as two doses at the beginning and at 8 weeks, in a 16 week duration trial. Kendrick et al. [32] compared cholecalciferol 2000 IU with calcitriol 0.5 μg daily for 6 months and did not detect any change in FMD.

There is a lack of interventional vitamin D studies with enough power to investigate hard endpoints. Instead, surrogate markers of cardiovascular risk have been used, such as pulse wave velocity and pulse wave-form analysis (PWV/PWA) and flow mediated vasodilatation (FMD) [21, 22, 33, 34]. Whereas PWV/PWA are complex measures of both beta-2 induced vasodilation [34], arterial stiffening and calcification [3], FMD is primarily a measure of the capacity of the endothelial cells to produce Nitic Oxide (NO) [35]. Since FMD is a measure of function and not structure, it is an earlier sign of vascular disease, and likely easier to affect by shorter duration of treatment. Even so, PWV and FMD are interrelated [36] and both are predictors of cardiovascular risk [6, 22, 33, 34, 37].

There seem to be a discrepancy between the results for PWV/PWA and FMD after treatment with vitamin D compounds to CKD patients. The reasons are probably many, but one important factor might be, that PWV/PWA is also a measure of arterial remodelling. Although probably due to inflammation in the first place [7], when established the structural changes in the vasculature are likely harder to affect and reverse. CKD patients have an accelerated remodelling with fibrosis and calcification [1, 3], and concerns have been raised [7] that we may intervene too late in the process in our attempts to ameliorate CKD associated vascular disease.

For these reasons we chose to use FMD as the only outcome. It is a more direct measure of NO availability, with clear antioxidant, anti-inflammatory and eNOS upregulating pathways for vitamin D actions [23–25].

We found 14 studies measuring the effect of vitamin D on different aspects of vascular function in CKD. Of these, 10 studied the effect on PWV and or PWAix with 26–120 participants, duration of 8 to 44 weeks, with CKD stage 3–5 and various vitamin D compounds and doses. Three studies reported positive effects on PWV [38–40], the rest did not detect any change after treatment. Two articles [41, 42] assessed iontophoresis by acetylcholine showing ameliorated microvascular function with treatment, though interpreted with caution due to the small number of patients and short duration. One study investigated reactive hyperaemia index, with no significant change in treated patients [43].

We chose a fixed model statistical analysis as our primary model. The reason was the few and small studies performed, which makes the results hard to generalize and thus indicated the use of a fixed model [27]. Even so, the random model performed as a secondary analysis also showed significant results of vitamin D intervention on FMD, which may allow generalization of our results.

There was, not surprisingly, a substantial heterogeneity in the fixed model. There were too few studies to perform a meta-regression of significant cofactors, but when the studies were inspected for clinical heterogeneity there were some important differences. The largest study [38] had the strongest effect size and the youngest population, originating from India, while the other populations were from western countries. In this study cholecalciferol was used as treatment in comparison with paricalcitol 1 to 2 μg in the other studies. This study also had the longest duration (16 weeks), and the highest Jadad score (5p). There was also a difference in baseline 25OH-vitamin D with significantly lower levels in the two studies with the strongest effect sizes [38, 44]. Theti et al. [45], who failed to show significant results, included only patients with diabetic nephropathy, in contrast to Kumar et al. [38] and Lundwall et al. [42] who excluded diabetics.

Even though the number of studies were too few for subgroup analyses, it is interesting to discuss the fact that the two studies that did not show positive effect of treatment with paricalcitol both used 1 μg [42, 45], in comparison with the others using 2 μg. One of them, Theti et al., [45] was also the only one investigating the effects on diabetic nephropathy. Highly interesting is also the fact that the study with the strongest effect size [38], used inactive treatment with cholecalciferol, to patients substantially younger than in the other studies, but in the same CKD stage (3–4).

Of note, there seem to be a tendency to less negative effects on calcium and phosphate metabolism with treatments using precursors of vitamin D [11–14, 46]. This might be favourable in treating vascular disease, and is also supported by a study of Zoccali et al. [47]. They saw, in a sub-study of the PENNY trial [44], that the effect of paricalcitol on FMD was most pronounced in patients with no change in phosphate during the study, and abolished in patients with the highest rise in phosphate levels, indicating the importance of different aspects of CKD mineral and bone disorders. These results might imply the use of phosphate binders in combination with active vitamin D.

## Conclusion

Even though our results are hard to generalize due to the small number of studies and patients included, we show favourable effects in both the fixed and the random model, suggesting benefits of vitamin D intervention on endothelial function. Our results also indicate that the highest impact is seen in younger patients, probably due to an earlier stage of disease, where vascular remodelling has not yet been established. It might also be more favourable with the precursor than with active treatment, especially since there seemed to be a need of high doses of active compounds for effects. This is possibly due to less increased calcium and phosphate levels with inactive treatment. There is still a great need for larger and longer studies on this topic, to a proper selection of CKD patients at earlier stages of their vascular disease, and with sufficient power to assess hard endpoints.

## Additional files

Additional file 1: (SearchCochrane): Data search Cochrane; Data search strategy for Cochrane reviews and Cochrane trials. (DOCX 18 kb)

Additional file 2: (Search Pubmed): Data search PubMed/Medline; Data search strategy for PubMed/Medline (DOCX 24 kb)

Additional file 3: (Web of Science, Embase): Data search Web of Science and Embase; Data search strategy for Web of Science and Embase. (DOCX 128 kb)

Additional file 4: (search software): Additional search information; Includes details about search software used, and special features used in the different searches. (DOCX 66 kb)

Additional file 5: (MAdataBMCN): Individual study data; Includes an excel file with the data used to perform the statistical part of the meta-analysis. (XLSX 50 kb)

## Abbreviations

ACE-i: angiotensin converting enzyme inhibitor
ARB: Angiotensin receptor blocker
CI: confidence interval
CKD: chronic kidney disease
CV: Cardiovascular
ESC: European Society of Cardiology
Echo: Echocardiography
FMD: flow mediated vasodilation
hPTH: hyperparathyreoidism
ITT: intention to treat
KDIGO: Kidney Disease Improving Global Outcome
MBD: Mineral bone disorder
NO: Nitic Oxide
PWA: Pulse wave-form analysis
PWV: Pulse wave velocity
RCTs: Randomized controlled trials
SD: standard deviation
SE: Standard error
STANDmean ES: standardized mean difference effect size
WoS: Web of Science
