# Supplementary material for: Treating endothelial dysfunction with vitamin D in chronic kidney disease: a meta-analysis
Source: BMC Nephrol. 2018 Sep 25;19:247. doi: 10.1186/s12882-018-1042-y (PMC6156877; doi:10.1186/s12882-018-1042-y)
Supplement: Supplementary file 1 — (SearchCochrane): Data search Cochrane; Data search strategy for Cochrane reviews and Cochrane trials. (DOCX 18 kb) [file 12882_2018_1042_MOESM1_ESM.docx]

Systematic search in Cochrane

**Population:** Chronic kidney disease

**Intervention:** Treatment with vitamin D

**Control:** Placebo

**Outcome:** Flow mediated vasodilation

**Study type**: Randomized trials.

**Filter**: Published 2000-01-01 or later.

| **Search P: Chronic kidney disease** | | | |
| --- | --- | --- | --- |
| **Search** | **Terms** | **Nr** | **Comments** |
| #1 | MeSH descriptor: [Kidney Failure, Chronic] explode all trees | 3978 |  |
| #2 | chronic kidney fail* or chronic renal fail* or endstage renal* or end-stage renal* or end stage renal* or endstage kidney* or end-stage kidney* or end stage kidney* | 12470 |  |
| #3 | MeSH descriptor: [Renal Insufficiency, Chronic] this term only | 931 |  |
| #4 | MeSH descriptor: [Kidney Diseases] this term only | 1767 |  |
| #5 | chronic | 100462 |  |
| #6 | #4 and #5 | 546 |  |
| #7 | Chronic renal* or Chronic kidney* | 15067 |  |
| #8 | #3 or #4 or #6 or #7 | 16288 |  |
| #9 | endstage or end-stage or end stage* | 17399 |  |
| #10 | #8 and #9 | 3509 |  |
| #11 | Ckf or crf or esrd or eskd or esrf or eskf or esri | 2298 |  |
| #12 | #1 or #2 or #10 or #11 | 13176 |  |

| **Search I: vitamin D** | | | |
| --- | --- | --- | --- |
| Search | Seach terms | Nr | Comments |
| #13 | vitamin d or vitamin d2 or vitamin d3 or cholecalciferol* or colecalciferol* or hydroxycholecalciferol* or hydroxycolecalciferol* or dihydroxycholecalciferol* or dihydrotachysterol* or maxacalcitol or oxacalcitriol or paricalcitol or doxercalciferol or dihydroxyvitamin* or falecalcitriol or calcitriol* or alfacalcidol* or alphacalcidol* or calcifediol* or calciphediol* or calcipotriol* or calcipotriene or Epicalcitriol or Lexacalcitol or seocalcitol or tacalcitol or ergocalciferol* | 12666 |  |
| #14 | MeSH descriptor: [Vitamin D] explode all trees | 3113 |  |
| #15 | #13 or #14 | 12668 |  |

| **Search study type: Randomized trials** | | | |
| --- | --- | --- | --- |
| **Search** | **Search terms** | **Nr** | **Comments** |
| #16 | MeSH descriptor: [Randomized Controlled Trial] explode all trees | 192 |  |
| #17 | MeSH descriptor: [Controlled Clinical Trial] this term only | 35 |  |
| #18 | random* | 779361 |  |
| #19 | #16 or #17 or #18 | 779366 |  |

| **Combined search** | | | |
| --- | --- | --- | --- |
| **Search** | **Search terms** | **Nr** | **Comments** |
| #1 | P | 13176 |  |
| #2 | I | 12668 |  |
| #3 | Studietyp | 779366 |  |
| **#4** | **#1 AND #2 AND #3** | **919** |  |
| **#5** | **#4 Filter: Published 2000-2018** | **300** |  |
