# Supplementary material for: Treating endothelial dysfunction with vitamin D in chronic kidney disease: a meta-analysis
Source: BMC Nephrol. 2018 Sep 25;19:247. doi: 10.1186/s12882-018-1042-y (PMC6156877; doi:10.1186/s12882-018-1042-y)
Supplement: Supplementary file 2 — (Search Pubmed): Data search PubMed/Medline; Data search strategy for PubMed/Medline (DOCX 24 kb) [file 12882_2018_1042_MOESM2_ESM.docx]

Systematic search in Pubmed

**Population:** Chronic kidney disease

**Intervention:** Treatment with vitamin D

**Control:** Placebo

**Outcome:** Flow mediated vasodilation

**Study type**: Randomized trials.

**Filter**: Published 2000-01-01 or later

| **Search I: vitamin D** | | | |
| --- | --- | --- | --- |
| **Search** | **Search terms** | **Nr** | **Comments** |
| #1 | Vitamin D[Mesh] | 52677 |  |
| #2 | vitamin d[tw] OR vitamin d2[tw] OR vitamin d3[tw] | 67159 | Truncation or [all] is too broad |
| #3 | cholecalciferol* OR  colecalciferol* | 7712 | Truncation rules out mesh |
| #4 | hydroxycholecalciferol* OR hydroxycolecalciferol* | 4505 |  |
| #5 | dihydroxycholecalciferol* | 3164 | Dihydroxycolecalciferol is not in PubMed |
| #6 | dihydrotachysterol* | 785 |  |
| #7 | maxacalcitol | 296 |  |
| #8 | oxacalcitriol | 134 |  |
| #9 | paricalcitol | 612 |  |
| #10 | doxercalciferol | 145 |  |
| #11 | dihydroxyvitamin* | 12500 |  |
| #12 | falecalcitriol | 64 |  |
| #13 | calcitriol* | 19946 |  |
| #14 | alfacalcidol* OR alphacalcidol* | 1242 |  |
| #15 | calcifediol* OR calciphediol* | 3680 |  |
| #16 | calcipotriol* | 872 |  |
| #17 | calcipotriene | 973 |  |
| #18 | Epicalcitriol | 2 |  |
| #19 | Lexacalcitol | 2 |  |
| #20 | seocalcitol | 182 |  |
| #21 | tacalcitol | 243 |  |
| #22 | ergocalciferol* | 3233 |  |
| ***#23*** | ***#1 OR #2 OR #3 OR #4 OR #5 OR #6 OR #7 OR #8 OR #9 OR #10 OR #11 OR #12 OR #13 OR #14 OR #15 OR #16 OR #17 OR #18 OR #19 OR #20 OR #21 OR #22*** | ***80007*** |  |

| **Search P: Chronic kidney disease** | | | |
| --- | --- | --- | --- |
| Search | Search terms | Nr | Comments |
| #1 | Kidney Failure, Chronic[Mesh] | 87197 |  |
| #2 | chronic kidney fail* | 87466 | At trunkation the words are interpreted as a phrase. Automapping is not performed |
| #3 | chronic renal fail* | 22737 | Same as above. |
| #4 | endstage renal* OR end-stage renal* OR end stage renal* | 34165 | Endstage renal disease is an entry term for meSH-term “Kidney Failure, Chronic”. |
| #5 | endstage kidney* OR end-stage kidney* OR end stage kidney* | 2643 | Endstage kidney disease is an entry term for meSH-term “Kidney Failure, Chronic”. |
| #6 | Renal insufficiency, chronic[mesh] | 101479 |  |
| #7 | "Kidney Diseases"[Mesh:NoExp] AND chronic | 14715 |  |
| #8 | Chronic renal* | 32403 |  |
| #9 | Chronic kidney* | 42364 |  |
| #9 | #6 OR #7 OR #8 OR #9 | 140913 |  |
| #10 | endstage OR end-stage OR end stage* | 61180 |  |
| #11 | #9 AND #10 | 24960 |  |
| #12 | Ckf OR crf OR esrd OR eskd OR esrf OR eskf OR esri | 117725 | A nr of abbreviations are applied that contibutes with approx 8000 articles. |
| ***#13*** | ***#1 OR #2 OR #3 OR #4 OR #5 OR #11 OR #12*** | ***133582*** |  |

| **Search study type: Randomized trials** | | | |
| --- | --- | --- | --- |
| **Search** | **Search terms** | **Nr** | **Comments** |
| #1 | randomized controlled trial[pt] | 456386 |  |
| #2 | controlled clinical trial[pt] | 543793 |  |
| #3 | Randomized Controlled Trials as topic[mesh] | 116829 |  |
| #4 | Controlled Clinical Trials as Topic[Mesh:NoExp] | 5307 | NoExp excludes underlying terms |
| #5 | Clinical Trials as Topic[mesh:NoExp] | 183004 | NoExp excludes underlying terms |
| #6 | Random allocation[mesh] | 93570 |  |
| #7 | randomized OR randomised | 827647 |  |
| #8 | randomization OR randomisation | 124765 |  |
| #9 | randomly | 287385 |  |
| #10 | rct | 16615 |  |
| #11 | placebo | 207206 |  |
| ***#12*** | ***#1 OR #2 OR #3 OR #4 OR #5 OR #6 OR #7 OR #8 OR #9 OR #10 OR #11*** | ***1243412*** |  |

| **Combined search** | | | |
| --- | --- | --- | --- |
| **Search** | **Search terms** | **Nr** | **Comments** |
| #1 | P | 119341 |  |
| #2 | I | 79631 |  |
| #3 | Study type | 1216416 |  |
| **#4** | **#1 AND #2 AND #3** | **401** |  |
| **#5** | **#4 Filter: Published 2000-2018** | **299** |  |
