# Supplementary figures and images for: Treating endothelial dysfunction with vitamin D in chronic kidney disease: a meta-analysis
Source: BMC Nephrol. 2018 Sep 25;19:247. doi: 10.1186/s12882-018-1042-y (PMC6156877; doi:10.1186/s12882-018-1042-y)

Web of Science


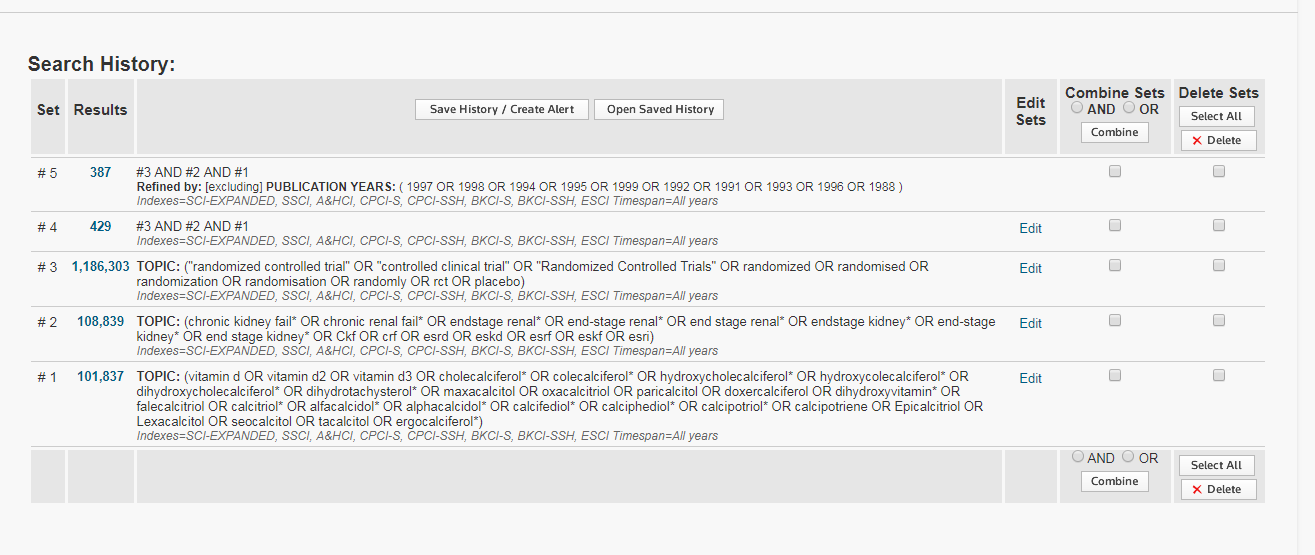


Embase


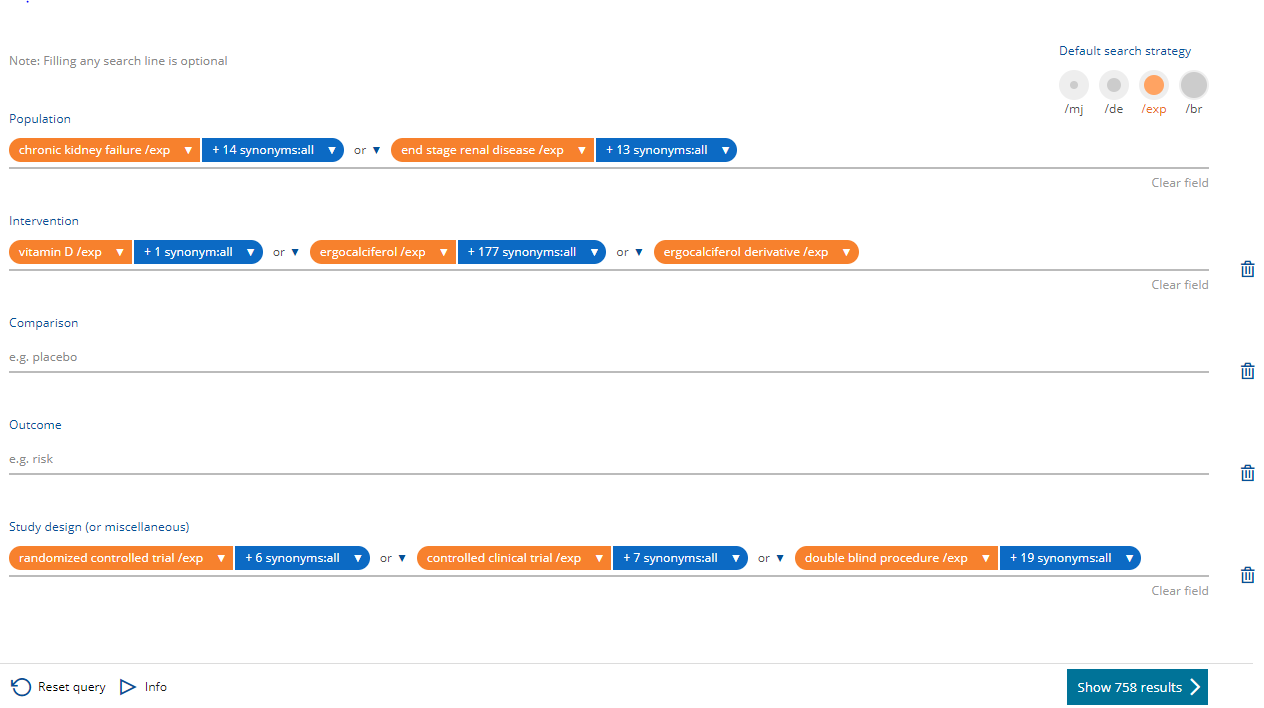

Supplement: Supplementary file 3 — (Web of Science, Embase): Data search Web of Science and Embase; Data search strategy for Web of Science and Embase. (DOCX 128 kb) [file 12882_2018_1042_MOESM3_ESM.docx]
