# Supplementary material for: Treating endothelial dysfunction with vitamin D in chronic kidney disease: a meta-analysis
Source: BMC Nephrol. 2018 Sep 25;19:247. doi: 10.1186/s12882-018-1042-y (PMC6156877; doi:10.1186/s12882-018-1042-y)
Supplement: Supplementary file 4 — (search software): Additional search information; Includes details about search software used, and special features used in the different searches. (DOCX 66 kb) [file 12882_2018_1042_MOESM4_ESM.docx]

SEARCH SOFTWARE INFORMATION:

PubMed: we used the only available version, with free access online via [https://www.ncbi.nlm.nih.gov/pubmed/](https://www.ncbi.nlm.nih.gov/pubmed/). It uses the thesaurus MeSH and explode MeSH-terms automatically.  In one case we chose to use the addition [Mesh:NoExp]. This was used to exclude kidney diseases that was not applicable for the research question (we wanted patients with the diagnosis of chronic kidney disease, from any cause and in any stage).

Embase: We used the software from Elsevier (the one that Karolinska Institute is using). It has the thesaurus Emtree where you decide if explosion should be used or not. As for PubMed explosion was used in all cases but one, for the same reason. We also included synonyms to make sure all substance- and label names for vitamin D were included.

Web of Science: We used the only available software from Clarivate. There is no thesaurus for this software and therefore no possibility to explode terms.

Cochrane: We used the only available software from Wiley for Cochrane Library. It has ”explode all trees” as default when searching with MeSH-terms, and as for PubMed and Embase this was not used in one case, for the same reason as above. When searching in free text it is set per default to search for alternative word variations, which was then also performed.

The references were collected in an EndNote library (version X8). EndNote is produced by Clarivate. We used the function to clear all duplicates from the different databases.
